# Morphological phenotyping of the aging cochlea in inbred C57BL/6N and outbred CD1 mouse strains

**DOI:** 10.1111/acel.14362

**Published:** 2024-10-31

**Authors:** Chiara Attanasio, Antonio Palladino, Daniela Giaquinto, Ferdinando Scavizzi, Marcello Raspa, Chiara Peres, Camilla Anastasio, Paola Scocco, Carla Lucini, Paolo de Girolamo, Livia D'Angelo, Elena De Felice

**Affiliations:** ^1^ Department of Veterinary Medicine and Animal Production University of Naples Federico II Naples Italy; ^2^ Department of Agricultural Sciences University of Naples Federico II Naples Italy; ^3^ National Research Council, CNR—Institute of Cellular Biology and Neurobiology Monterotondo Italy; ^4^ National Research Council, CNR—Institute of Biochemistry and Cell Biology ‐ International Campus EMMA‐INFRAFRONTIER‐IMPC Monterotondo Italy; ^5^ Department of Precision Medicine University of Campania Luigi Vanvitelli Naples Italy; ^6^ School of Biosciences and Veterinary Medicine University of Camerino Camerino Italy

**Keywords:** aging cochlea, animal models, morphological phenotyping, mouse strains, sensorineural hearing loss

## Abstract

Morphological mouse phenotyping plays a pivotal role in the translational setting and even more in the area of auditory research, where mouse is a central model organism due to the evolutionary genetic relationship and morpho‐functional analogies with the human auditory system. However, some results obtained in murine models cannot be translated to humans due to the inadequate description of experimental conditions underlying poor reproducibility. We approach the characterization of the aging process of the mouse cochlea in animals up to 18 months of age belonging to two of the most used outbred (CD1) and inbred (C57BL/6N) strains. Striving to reduce any environmental variable we performed our study compliantly to the ARRIVE guidelines. We integrated instrumental data (auditory brainstem response test), with morphological analyses to correlate functional discrepancies to morphological changes and track the differences in the evolution of sensorineural hearing loss in the two strains. We featured the localization of Gipc3, Myosin VIIa, and TMC1 in hair cells of the Corti organ as well as NF 200 and the density of type I neuron in the spiral ganglion. We outlined age‐related hearing loss (ARHL) in both strains, and a clear drop in the selected marker localization. However, in CD1 we detected a different trend allowing the identification of potential strain‐specific mechanisms, namely an increase in myosin VIIa in 6 months aging mice in comparison to 2 months old animals. Our findings represent an asset to investigate the strain‐dependent physiological trigger of ARHL providing new insights in the translational area.

## Graphical abstract

This study examines age‐related auditory decline in two mouse strains, CD1 and C57BL/6N, by correlating auditory brainstem response tests with morphological analyses. The findings suggest potential strain‐specific protective mechanisms, involving Myosin VIIa in CD1 mice, and highlight differences in spiral ganglion neuron loss. The study emphasizes the importance of selecting appropriate mouse strains for auditory research and offers a protocol for preserving cochlear anatomy during analysis.

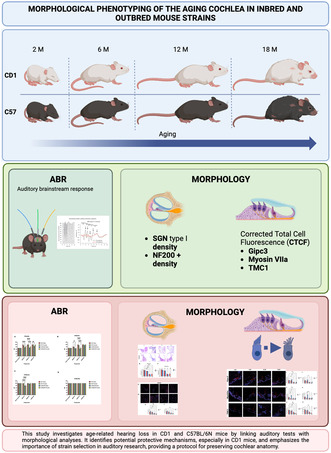

**Abbreviations**
ABR: auditory brainstem response
ARHL: age‐related hearing loss
CTCF: Corrected Total Cell Fluorescence
ER: endoplasmic reticulum
Gipc3: GAIP‐interacting protein C‐terminus
HCs: hair cells
H‐E: Hematoxylin/Eosin
MT: mechanotransduction
NF200: Neurofilament protein NF200
PB: phosphate buffer
PBS: phosphate‐buffered saline
ROI: region of interest
SGNs: spiral ganglion neurons
SNHL: sensorineural hearing loss
SPF: Specific Pathogen‐Free
TMC1: Transmembrane channel‐like protein 1

## 1 INTRODUCTION

The world report on hearing released in 2021 predicts that around 2.5 billion people, 1 out of 4 people worldwide, will be affected by some degree of hearing loss by 2050. Different type of age‐related hearing loss (ARHL) has been identified and linked to different anatomical structures, namely sensory hair cells (HCs), stria vascularis and auditory nerve fibers leading to sensory, strial, and neural hearing loss respectively. From these types of ARHL frequently derive forms of hypoacusia induced by the alteration of multiple structures among which that defined sensorineural (SNHL) is the most common and is due to degeneration of the sensory HCs in the organ of Corti and spiral ganglion neurons (SGNs), their innervating afferent neurons.

The study of age‐related cochlear disease in humans is extremely challenging due to the limited availability of cochlear tissue, the difficulty and expense to perform longitudinal studies as well as the impact of genetic and environmental factors which may affect data interpretation (Castaño‐González et al., 2024; Wang & Puel, 2020). Therefore, the study of age‐related cochlear pathology is crucially dependent on the use of animal models (Castaño‐González et al., 2024). In the arena of auditory research, mouse is a central model organism due to a high degree of evolutionary genetic relationship and morpho‐functional analogies with the human auditory system (Bowl & Dawson, 2015; Sindura & Banerjee, 2019). SNHL in mice reveals histopathological alterations like those reported in humans such as decreased number and degeneration of sensory HCs and SGNs as well as structural abnormalities of the organ of Corti architecture (Fetoni et al., 2022; Henry & Chole, 1980; Jyothi et al., 2010).

However, some results gained in murine models are not translatable to humans. In many research papers, indeed, details about origin, husbandry, and procedures are not adequately described to facilitate data reproducibility and translatability (Justice & Dhillon, 2016). For this reason, it is useful to follow specific guidelines reporting the best practice to increase result reproducibility in animal studies. Since habitat and microbiome affect experimental outcome the use of animals from internal colonies, instead of animals purchased from external suppliers, along with experimenters blinded to the experimental conditions may be helpful in limiting data misinterpretation (Justice & Dhillon, 2016; Nguyen et al., 2015). For many decades, isogenic mouse strains, usually known as inbred, were the most used mouse models, due to the assumption that by removing the individual genetic variability the phenotypic one would have been reduced. However, inbred strains are not always the golden standard in the translational setting, for example in the case of genetic‐based disease studies in which their adequacy can only be partial considering the genetic diversity of human populations (Tuttle et al., 2018). Considering that the age‐dependent SNHL often starts with hair cell and continues with afferent neurons degeneration (Liberman & Kujawa, 2017), our target was to identify the difference in morphological alterations triggering these events in an inbred and outbred strain to provide a valuable framework to link basic research to preclinical applications. Few studies have been performed concerning the localization of specific markers in HCs of adult mice (Kurima et al., 2015; Mahendrasingam et al., 2017; Wang et al., 2015; Xiong et al., 2012; Xu et al., 2023), although they provide significant data to unravel both function and regulation of specific proteins (Li et al., 2019).

We approached the characterization of aging process in the cochlea of mice belonging to two of the most commonly used outbred (CD1) and inbred (C57BL/6) strains by coupling instrumental data performed via auditory brainstem response test (ABR) (Paciello et al., 2021; Willott, 2006) with morphological analyses on the same individuals.

Our experimental design enabled us to correlate functional discrepancies with morphological changes between the two strains over aging identifying strain‐dependent differences in age‐related hallmarks in animals ranging from 2 to 18 months of age. Specifically, we identified GAIP‐interacting protein C‐terminus (Gipc3), Myosin VIIa, and Transmembrane channel‐like protein 1 (TMC1) in the HCs. In particular, Gipc3 localizes throughout the cytoplasm of IHCs and OHCs but not at the stereocilia bundle (Charizopoulou et al., 2011), Myosin VIIa is an actin‐binding mechanoenzyme localized in stereocilia but also throughout the inner and outer HC cytoplasm (Sirko & Kozlov, 2022; Wolfrum et al., 1998) while TMC1, as part of the hair cell mechanotransduction channels, localizes, exclusively or non‐exclusively, in the stereocilia (Corey et al., 2019). Further, to gain more insights on the correlation between fiber degeneration and sensory hair cell loss we labelled neural cytoskeleton by NF200 (Liberman & Kujawa, 2017).

In addition, we provided an effective decalcification protocol to study the morphological features of adult mouse cochlea guaranteeing the integrity of its anatomy and microarchitecture.

## 2 METHODS

### 2.1 Animals

The study was held at CNR (Italian National Research Council)‐EMMA (European Mouse Mutant Archive), a member of the International Mouse Phenotyping Consortium (n.d.; www.mousephenotype.org) using mice belonging to the internal colonies and compliantly with the ARRIVE guidelines (Table S1) (Percie du Sert et al., 2020).

The experimental procedures were agreed upon, reviewed and approved by local animal welfare oversight bodies (CNR), the experiments were performed with the approval and direct supervision of the CNR‐IBCN/Infrafrontier‐Animal Welfare and Ethical Review Body (AWERB), in accordance with general guidelines regarding animal research, and in compliance with the Legislative Decree 26/2014, transposing the 2010/63/EU Directive. The protocol was approved by the Italian Ministry of Health (1177/2020‐PR).

At CNR‐EMMA mice were housed in the Specific Pathogen‐Free (SPF) barrier unit (Monterotondo Scalo, Rome, Italy). Mice at 2, 6, 12, and 18 months of age of both sexes belonging to C57BL/6N (*n* = 64) and CD1 (*n* = 64) strains were used in the study (*n* = 128 in total: *n* = 8 per time point/per sex/per strain).

The number of animals for each group was calculated using G‐Power 3.1.9.4 software for a repeated measures Anova test set as follows: mean effect size 0.25, significance level (*α*) 0.05, test power 0.80.

At birth, animals were randomly assigned to one of the four groups and used at the established timepoints. Mice were housed in individually ventilated cage systems (Tecniplast, Buguggiate, VA, Italy) set with temperature 21°C ± 2°C, relative humidity 55% ± 15%, air changes per hour 50–70, and controlled (12:12 h) light–dark cycle (7 am–7 pm). Mice had ad libitum access to water and standard rodent diet (Emma 23, Mucedola, Settimo Milanese, Italy). Environmental enrichment was guaranteed by equipping each cage with nesting paper and mouse houses. All procedures were performed under anesthesia, no adverse effects were expected, and therefore the study had no humanitarian endpoints. Any alterations were attributable to physiological aging and adequately recognized by trained and expert personnel. Exclusion criteria were based on the evaluation of body condition score, weight loss, reduced mobility, dehydration. If the animals had shown one or more of these signs they would have been excluded from the experiments. Animal care keepers, the surgeon, technicians, and outsourcing laboratory operators were not aware of the group allocation at the different stages of the experiment.

### 2.2 Auditory brainstem response

Each animal was tested at 2, 6, 12, and 18 months of age (*n* = 8 males and *n* = 8 females/strain/timepoint). Once sedated by intraperitoneal injection of a mixture of ketamine hydrochloride and medetomidine hydrochloride (80 mg/kg and 0.5 mg/kg body weight respectively), mice have been placed by aligning the center of the speaker with the external ear canal at a distance of 10 cm. The acoustic stimuli for ABR, *clicks* (100 μs duration) and *tone bursts*, 8, 16, 24 kHz, were generated by multi‐field magnetic speakers. To collect the bioelectrical potentials active, reference and ground electrodes were placed at forehead level, ventrolaterally to the left ear and over the tail respectively. Response to acoustic stimuli was recorded using BioSigRZ Software.

The ABR measurements were carried out within a soundproof room. The acquired responses were amplified, filtered (0.3–3 kHz), and averaged at each sound level (512 repeats of the same stimulus).

Hearing thresholds were determined offline as the Sound Pressure Level at which a I‐wave peak above background noise could be visually identified (0.1 μV) (Paciello et al., 2021).

### 2.3 Cochlea dissection

At each timepoint, 2, 6, 12, and 18 months mice belonging to CD1 and C57BL/6N strains, the same animals previously subjected to ABR, (*n* = 3/strain) were deeply anesthetized by intraperitoneal injection of ketamine hydrochloride and medetomidine hydrochloride (225 mg/kg of ketamine and 3 mg/kg of medetomidine respectively) and transcardially perfused with phosphate‐buffered saline (PBS) and 4% paraformaldehyde in 0.1 M phosphate buffer (PB) at pH 7.4 (Soriano‐Cantón et al., 2015).

**FIGURE 1:**
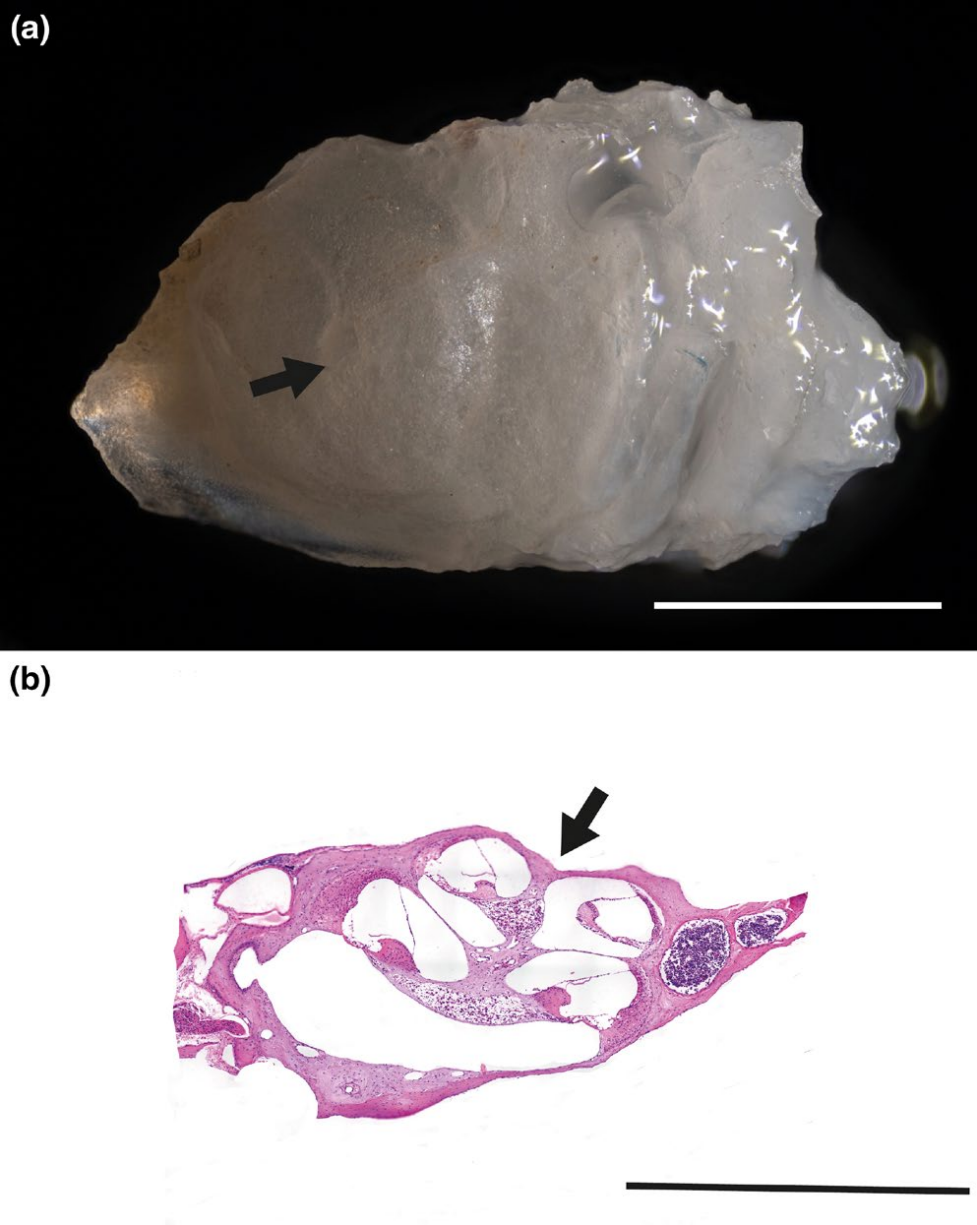
Dissected Cochlea: (a) shows the appearance of the dissected mouse cochlea, (b) displays the histological section of the tissue stained by Hematoxylin/Eosin (H‐E). Arrows indicate the modiulus. Scale bars 600 μm.

Afterwards, for cochleae collection heads were dissected and placed on a sterile pad. The skin of each head was then lifted and detached from the skull previously fixed by placing forceps into the orbital cavities. Once an incision has been made along the sagittal suture, a cut along the coronal suture was performed using a sharp blade (*n*. 11) taking care not to damage the cochlea. The brain was then carefully removed from the two halves of the skull for further analyses. The cochlea was identified within the temporal bone using a stereomicroscope, afterwards forceps were placed into the superior semicircular canal. To dissect all the tissues surrounding the cochlea, the organ was transferred into a Petri dish laying on ice and all the procedures were performed using a stereomicroscope (Miller et al., 2022). The temporal bone was carefully removed keeping the organ attached to the vestibule. The cartilaginous cochlear capsule was removed by inserting the tips of the forceps into the apex area or between the coils to expose the cochlear duct. The cochlea was then isolated after having been detached from the vestibular organ and the temporal bone using the forceps (Figure 1).

### 2.4 Tissue processing

Isolated cochleae (*n* = 3) were post‐fixed overnight (o/n) with 4% PFA at 4°C. They were, then, rinsed two times in PBS 1X and decalcified for 14 days in 2% EDTA (68 mM in 250 mL PBS) (Thermo Scientific, Geel, Belgium, product code J15701) at 4°C replaced every 2 days. The decalcification progress was monitored daily by inspecting the samples. Adequate decalcification was indicated by the samples becoming transparent and losing their bony consistency. Daily inspections involved observing the colour of the samples and performing a consistency test by applying gentle pressure with tweezer tips on the samples. Samples were considered sufficiently decalcified when they exhibited a spring‐back response after pressing.

After decalcification, cochleae were rinsed in PBS 1X, dehydrated in ethanol from 70% to 100%, treated with xylene (ROMIL, Cambridge, GB) and embedded in paraffin. Tissue slices 7 μm thick were cut by using a microtome (Thermo Scientific, Waltham, MA, USA).

#### 2.4.1 General histology

Sections were deparaffinized with xylene, then rinsed in ethanol from 100% to 70%, washed in water, and stained by Hematoxylin/Eosin (H/E) (Carlo Erba, Milan, Italy, product codes 446472/46634) for morphological analysis. The sections were mounted with Histomount Mounting Solution (Bio‐Optica, Milan, Italy, product code 600010001040101) and observed with a light microscope (Motic Panthera, Kowloon, Hong Kong). Images were acquired by a Leica DM6B microscope (Wetzlar, Germany).

#### 2.4.2 Immunofluorescence

Sections were rinsed in ethanol from 100% to 70%, washed in water and rinsed in Triton 0.1% (X‐100, Sigma, product code T9284) in PBS for 5 min. Non‐specific antigen binding was blocked using a solution of 1:33 normal goat serum in PBS/Triton for 30 min at room temperature (RT). Thereafter, sections were incubated with the primary antisera o/n at 4°C. The day after samples were washed in PBS 1X and incubated with a secondary antibody for 1 h at RT. Once rinsed in PBS/Triton nuclei were stained with DRAQ5 (1:500, Thermo Scientific, Geel, Belgium, prod. code 62251) and samples were observed using a Leica DM6B microscope. Non‐specific binding of the secondary antibody (Ab) to cellular components has been tested by incubating the tissue with the Ab diluent, without primary Ab. In Table 1 we reported the primary and secondary antibodies used.

**TABLE 1:** List of the primary and secondary antibodies used in the study.

Name	Host species	Product code	Company	Dilution
GIPC3	Rabbit	Orb183855	Biorbyt	1:100
Myosin VIIa	Rabbit	GTX23481	GeneTex	1:100
TMC‐1	Rabbit	PA5‐97128	Invitrogen	1:100
NF200	Rabbit	N4142	Sigma‐Aldrich	1:100
Alexa‐Fluor 555	Goat	Ab150078	ABCam	1:200

### 2.5 Quantitative image analysis

Type 1 SGNs were quantified by counting the nuclei detected in each ganglion section and measuring the ganglion area. Inclusion criteria were based on cell morphology and nuclear size (Carricondo & Romero‐Gómez, 2019; Jyothi et al., 2010). Cell density in each ganglion was calculated as number of cells/area ratio. Image analysis was performed by Image J (2.14.0/1.54f version).

Quantitative image analysis for GIPC3, Myosin VIIa, TMC‐1, and NF200 was performed by acquiring five images per sample by LSM 510 (Zeiss AG, Oberkochen, Germany) microscope were analyzed by ImageJ (2.14.0/1.54f version) (Koh et al., 2016; McCloy et al., 2014). In order to quantify the signal arising from the HCs specific markers (Gipc3, MyoVIIa, and TMC1) we used the Corrected Total Cell Fluorescence (CTCF) calculation method. This is a quantitative approach used to measure the fluorescence intensity of specific markers within cells, correcting for background fluorescence. The CTCF method involves several steps, starting with the acquisition of fluorescence microscopy images. To ensure reproducibility across the timepoints the confocal acquisition parameters within each experiment were set on 2 months samples as reference. Subsequently, on the images reconstructed by ImageJ, the region corresponding to cell of interest has been selected using drawing or selection tools in the image analysis software. The integrated density (the sum of the values of the pixels in the selected area), the area of the selected cell, and the mean fluorescence of the background (a region with no fluorescence) have been measured. The formula for CTCF is given by: CTCF = Integrated Density − (Area of selected cell × Mean fluorescence of background). This formula corrects the total fluorescence of the cell by subtracting the background fluorescence, calculated from a non‐fluorescent region near the cell. This step is crucial for obtaining accurate measurements of fluorescence intensity that are not influenced by non‐specific background signals. The CTCF method is particularly useful for this kind of studies, where precise quantification of fluorescence signals from specific cellular markers is essential for understanding cellular characteristics and functions. By analyzing multiple sections per animal, at least 5, we aimed to capture the variability and ensure the reliability of our fluorescence signal measurements across the organ of Corti hair cell population at different timepoints.

For quantifying NF200 positive cells, a distinct method was employed. Initially, the region of interest (ROI) was delineated as the spiral ganglion area using the “Freehand selection” tool in ImageJ. This area was then measured and marked. Subsequently, to determine cellular density, all NF200 positive cells with morphological characteristics resembling SGNs within the defined area were enumerated. The results have been then presented as positive cell density.

### 2.6 Statistical analysis

Auditory brainstem response analyses have been performed on eight animals of each sex and strain. Morphological analyses have been performed in triplicate. The graphics report data as mean ± standard deviation (SD). Differences between groups were analyzed by ANOVA followed by Tukey's HSD post‐hoc test with GraphPad Prism software 10.1.2 (324) version. *p* values <0.05 (*), *p* values <0.01 (**), *p* values <0.001 (***), and *p* values <0.0001 (****) have been considered statistically significant.

## 3 RESULTS

### 3.1 Differences in auditory brainstem response over aging

**FIGURE 2:**
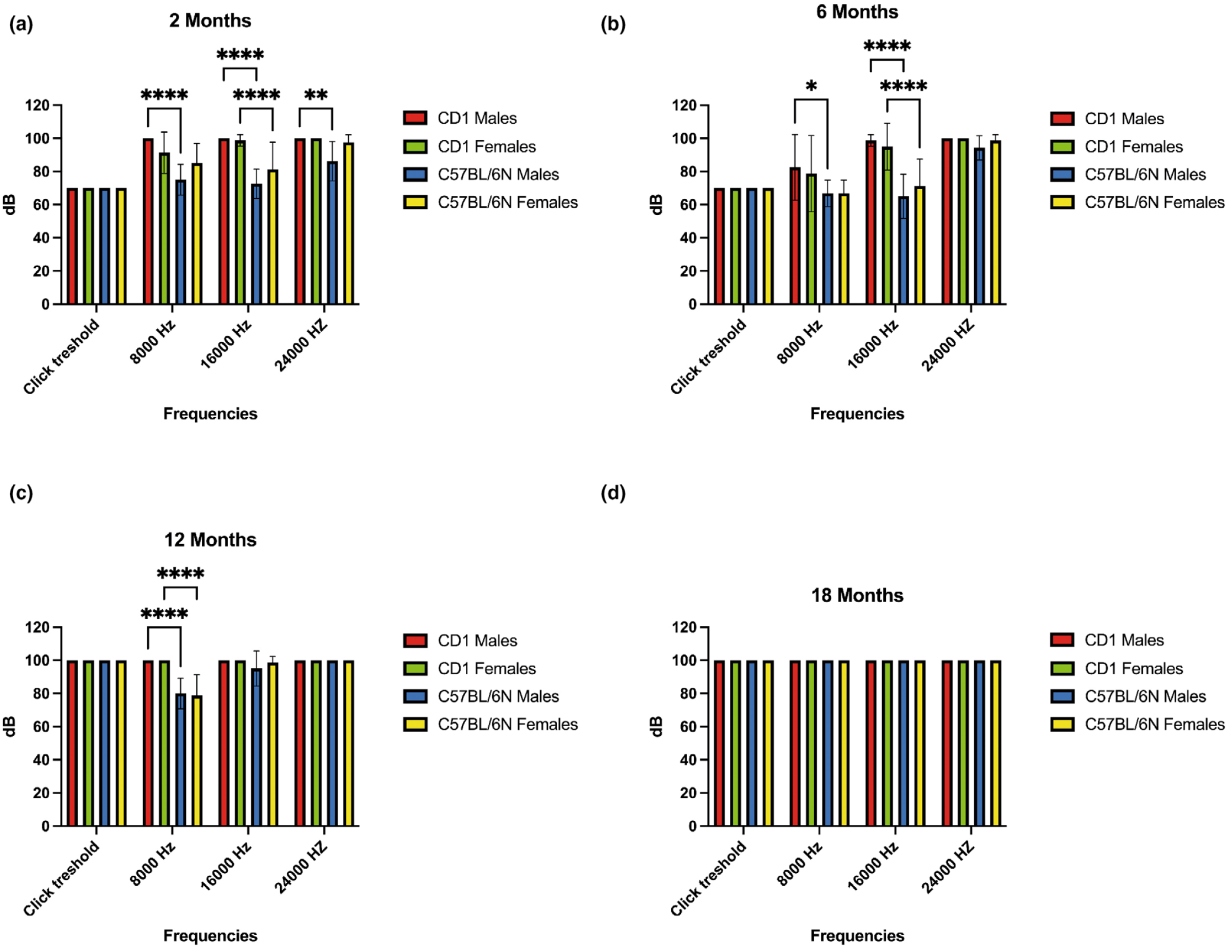
ABR results: The diagrams show the results of auditory brainstem response test in CD1 and C57BL/6N mice in both sexes at different ages: (a) 2 months, (b) 6 months, (c) 12 months, and (d) 18 months. Within the same diagram the results are grouped by type and frequencies of the acoustic stimuli (click threshold, 8000 Hz, 16,000 Hz and 24,000 Hz). Differences between groups were analyzed by ANOVA followed by Tukey's HSD post‐hoc test. *p* values <0.05 (*), *p* values <0.01 (**), and *p* values <0.0001 (****) have been considered statistically significant.

The ABR tests performed showed a similar trend in both strains and sexes at all the timepoints revealing that at 18 months of age all the animals were deaf with non‐evocative traces at 100 dB (Figure 2 and SI Figure S1). Acoustic perception, measured considering as reference points the time of appearance of the first and the fourth waves, and the distance first‐fourth waves, tends to decrease in young adults mice compared to young animals, except in the case of CD1 males which displayed already the lack of acoustic perception at 2 months of age at all the tested frequencies, low (8.000 Hz), intermediate (16.000 Hz) and high (24.000 Hz). In CD1 mice the acoustic threshold in young adult decreases of 17.5 dB and 12.5 dB in male and female animals respectively. These results indicate that in CD1 mice of both sexes at 6 months of age acoustic perception at low frequencies significantly improves in comparison to 2 months old animals and this datum in males is even clearer than in females.

More in details, there was a significant difference of tones stimuli (8, 16, and 24 kHz) thresholds during aging in the two strains. Comparing the sexes of the two strains, we noticed at low frequencies (8.000 Hz) a statistically significant difference between males at the 2 months of age. This difference was maintained at 6 and 12 months while in females it was displayed only at 12 months of age. At medium frequencies (16.000 Hz) we show a statistically significant difference in both males and females at 2 and 6 months of age while at high frequencies (24.000 Hz) there was a statistically significant difference only in 2 month old males (Figure 2).

### 3.2 Hair cell marker distribution over aging

We expressed the values in % to enable the comparison between the two strains since each sample has been acquired by setting acquisition parameters on sections belonging to animals of 2 months of age. Therefore, the data collected by measuring the relative fluorescence can be related to the single strain (SI Figure S2).

#### 3.2.1 Gipc3

**FIGURE 3:**
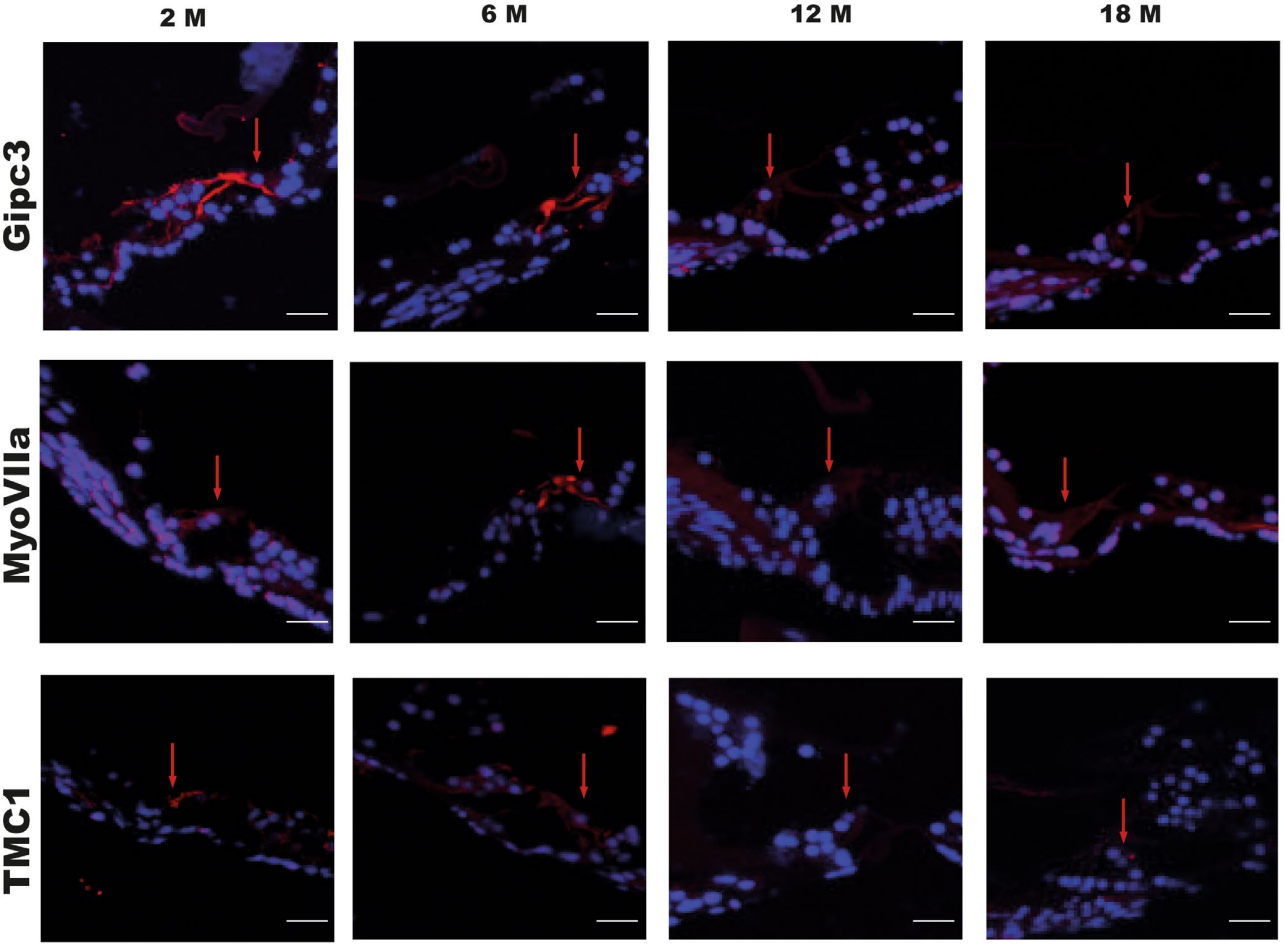
Hair cells specific markers: Immunofluorescence to Gipc3, MyoVIIa, and TMC1 in the organ of Corti of C57BL/6N mice at 2, 6, 12 and 18 months of age The images are representative of both the strains. Red arrows indicate inner hair cells in each section. Scale bars 20 μm.

**FIGURE 4:**
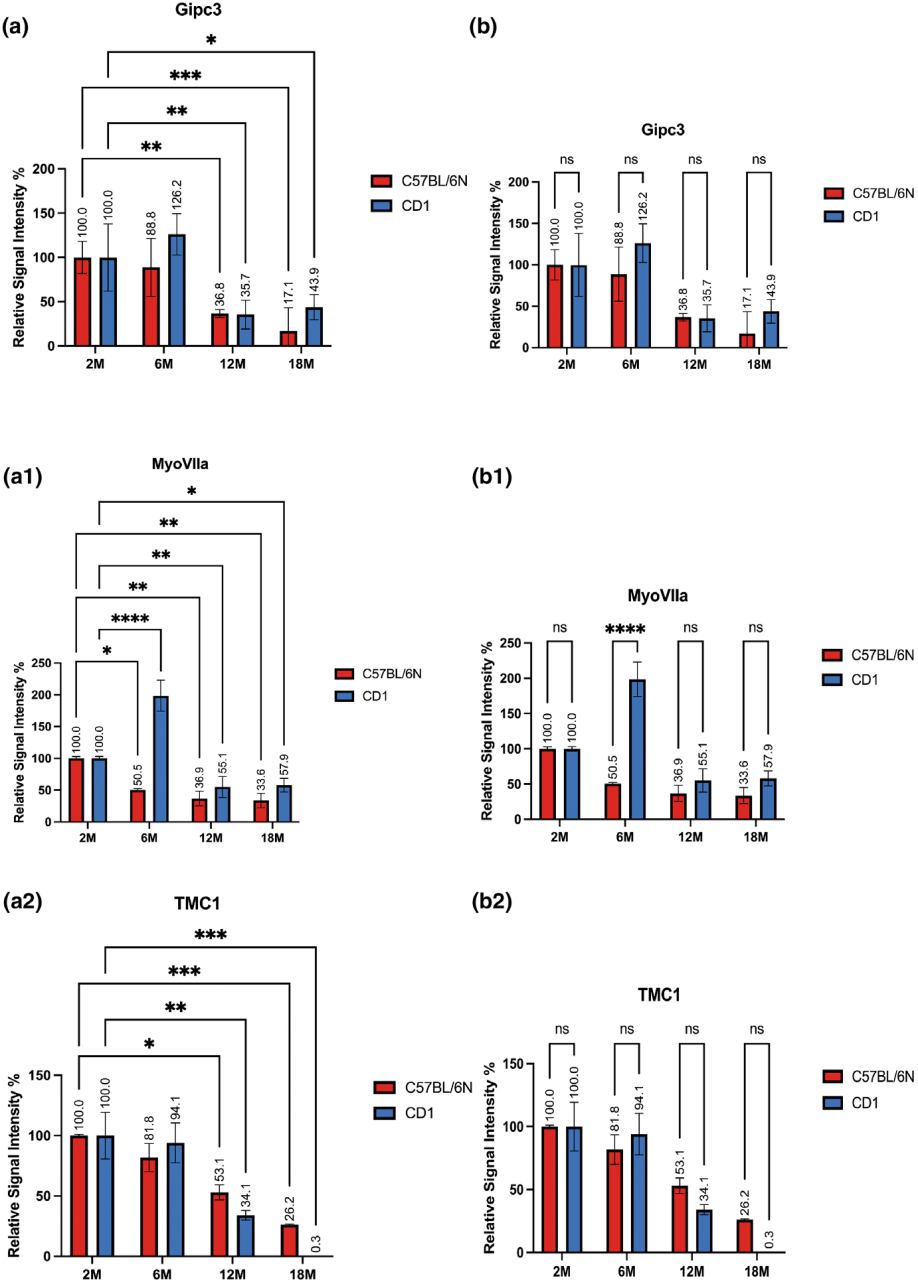
Hair cell markers quantitative image analysis: The histograms display the quantitative analysis of the immunofluorescence toGipc3, MyoVIIa, and TMC1 in hair cells. (a, a1, a2) Comparisons between the different timepoints, 2, 6, 12 and 18 months for each strain. (b, b1, b2) Comparisons between the two strains, C57BL/6N versus CD1, at each timepoint. Differences between groups were analyzed by ANOVA followed by Tukey's HSD post‐hoc test. *p* values <0.05 (*), *p* values <0.01 (**), *p* values <0.001 (***), and *p* values <0.0001 (****) have been considered statistically significant.

Via Gipc3 (GIPC PDZ Domain Containing Family, Member 3) immunostaining we marked the cytoplasm of both inner and outer HCs being this protein involved in the intracellular vesicle trafficking (Charizopoulou et al., 2011). Gipc proteins interact specifically with the C‐terminus of RGS‐GAIP (De Vries et al., 1998). Gipc3 localizes exclusively in cochlear HCs and SGNs, therefore, we detected its signal in the cytoplasm of both OHC and IHC with a higher intensity in mice of 2 and 6 months of age in both strains (Figure 3). We noticed a different trend over aging in the two strains. In C57B/6 N our data revealed a gradual decrease in signal intensity featured by a clear drop during the transition from 6 to 12 month of age, in details we reported a 11.2% and a 63.2% decrease in mice of 6 and 12 months in comparison to young mice respectively while between 12 and 18 month of age signal intensity displayed a reduction of 19.7%. In CD1 mice our data revealed an increase of 26.2% in OHCs and IHCs immunopositivity in mice at 6 months of age compared to younger mice, which means that GIPC3 in CD1 mice is more represented by 37.4% than in C57B/6 N mice of the same age. Still, in CD1 we reported a clear decline in signal intensity during the transition from young adult to adult stage (6–12 months) as witnessed by a 90.5% decrease. Also, a slight immunopositivity increase, of 8.2% was found in 18 months compared to 12 months aged mice. This difference, despite extremely low, confirms the variable trend of the data in CD1, probably due to the intrinsic variability of this outbred strain (Figure 4a). No statistically significant differences between the two strains were revealed by comparing the results at the different time points (Figure 4b).

#### 3.2.2 Myosin VIIa

Also in case of Myosin VIIa actin‐binding mechanoenzyme immunodetection we identified the inner and outer HC cytoplasm (Sirko & Kozlov, 2022; Zallocchi et al., 2012) (Figure 3). In C57B/6 N mice we observed a steady reduction in cell immunopositivity to this marker throughout the lifespan with values of 49.5%, 63.1% and 66.4% in mice of 6, 12 and 18 months of age in comparison to young animals. Conversely, CD1 6 months aged mice displayed a clear increase in signal intensity in comparison to 2 months aged animals as witnessed by a 93.9% value. Further, compliantly to this result, immunopositivity value dramatically dropped between 6 and 12 months of age when our analysis showed a signal intensity decrease by 44.9% in 12 months compared to young animals. A very slight immunopositivity increase, of 2.8% was found in 18 months compared to 12 months aged mice (Figure 4a1). A statistically significant difference between the two strains was reported only at 6 month of age (Figure 4b1).

#### 3.2.3 TMC1

Regarding TMC1 (Transmembrane channel‐like protein 1) we observed a sharp signal localized at the stereocilia bundles of the IHC in CD1 young animals, at 2 months of age, unlikely in C57B/6 N of the same age we found the same protein localization in OHC (Pan et al., 2013). Further, we detected a different localization, in the cytoplasm and in the perinuclear area, of this marker in both strains in adult and old animals, aging 12 and 18 months respectively (Figure 3). From the quantitative standpoint we observed an expected trend in both strains over the lifespan. In C57B/6 N HCs displayed a gradual immunoreactivity decrease as shown in both images and quantitative analysis. These latter displayed a decrease by 18.2%, 46.9%, and 73.8% in animals of 6, 12, and 18 months of age respectively in comparison to young mice. In CD1 mice, instead, we revealed very similar values in 2 and 6 months aged animals in comparison to what happens in the following life stages. In animals 6 month old, indeed, we observed just a 5.9% signal intensity decrease in contrast to a 65.9% and 99.7% reduction measured respectively in 12 and 18 months aged animals in comparison to young mice (Figure 4a2). No statistically significant differences between the two strains were revealed by the comparison of the different time points (Figure 4b2).

### 3.3 Spiral ganglion neuron

#### 3.3.1 H/E

**FIGURE 5:**
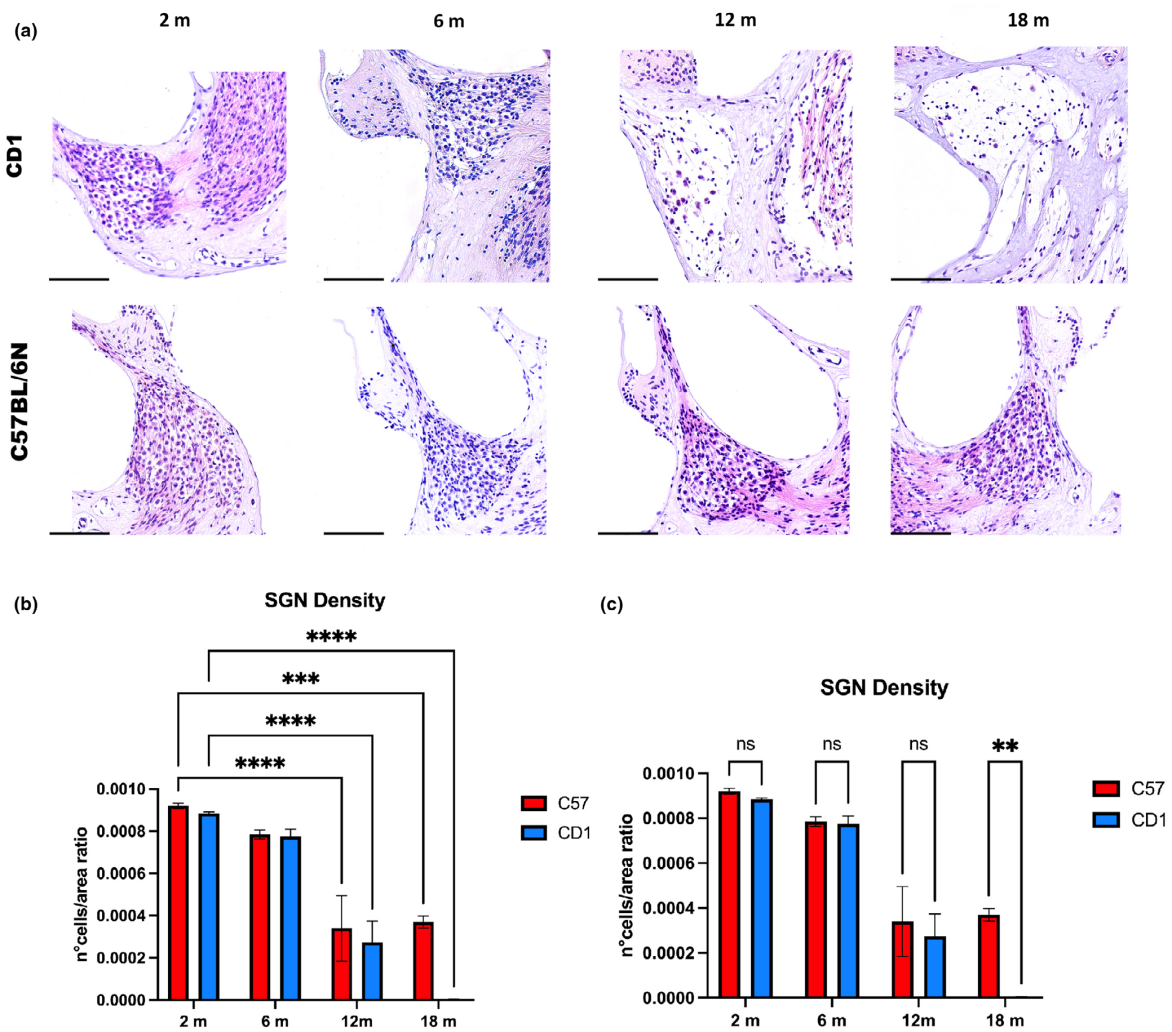
Spiral ganglion and neuronal density: (a) Hematoxylin/Eosin (H–E) staining of spiral ganglia of C57BL/6N and CD1 mice at 2, 6, 12, and 18 months of age. Scale bars 100 μm. (b, c) The histograms display the density of spiral ganglion neurons. (b) Comparisons between the different timepoints, 2, 6, 12 and 18 months for each strain. (c) Comparisons between the two strains, C57BL/6N versus CD1, at each timepoint. Data were analyzed by ANOVA followed by Tukey's HSD post‐hoc test. *p* values <0.01 (**), *p* values <0.001 (***), and *p* values <0.0001 (****) were considered statistically significant.

Over aging, the analysis of the histological sections showed a clear drop in the number of type 1 SGNs as evidently visible in the images of H‐E stained sections and in the graph displaying the quantitative data in both the strains (Figure 5). Over aging our analysis showed a gradual decrease in SGN number in both strains, however, a clear, statistically significant difference between the strains, has been revealed at 18 months when in CD1 SGNs become very scarce, around 1% of those detected in C57BL/6N (Figure 5b,c).

In addition, over aging we observed evident changes in cell morphology particularly related to cell shape, nuclear morphology and staining intensity. In particular, in 2 and 6 months aged animals the images display a well preserved cell shape both at cytoplasmic and nuclear level, with tight contacts among cells in both the strains in comparison to what happens in 12 and 18 months aged mice which also display a decreased number of SGNs (Figure 5a–c).

#### 3.3.2 NF200

**FIGURE 6:**
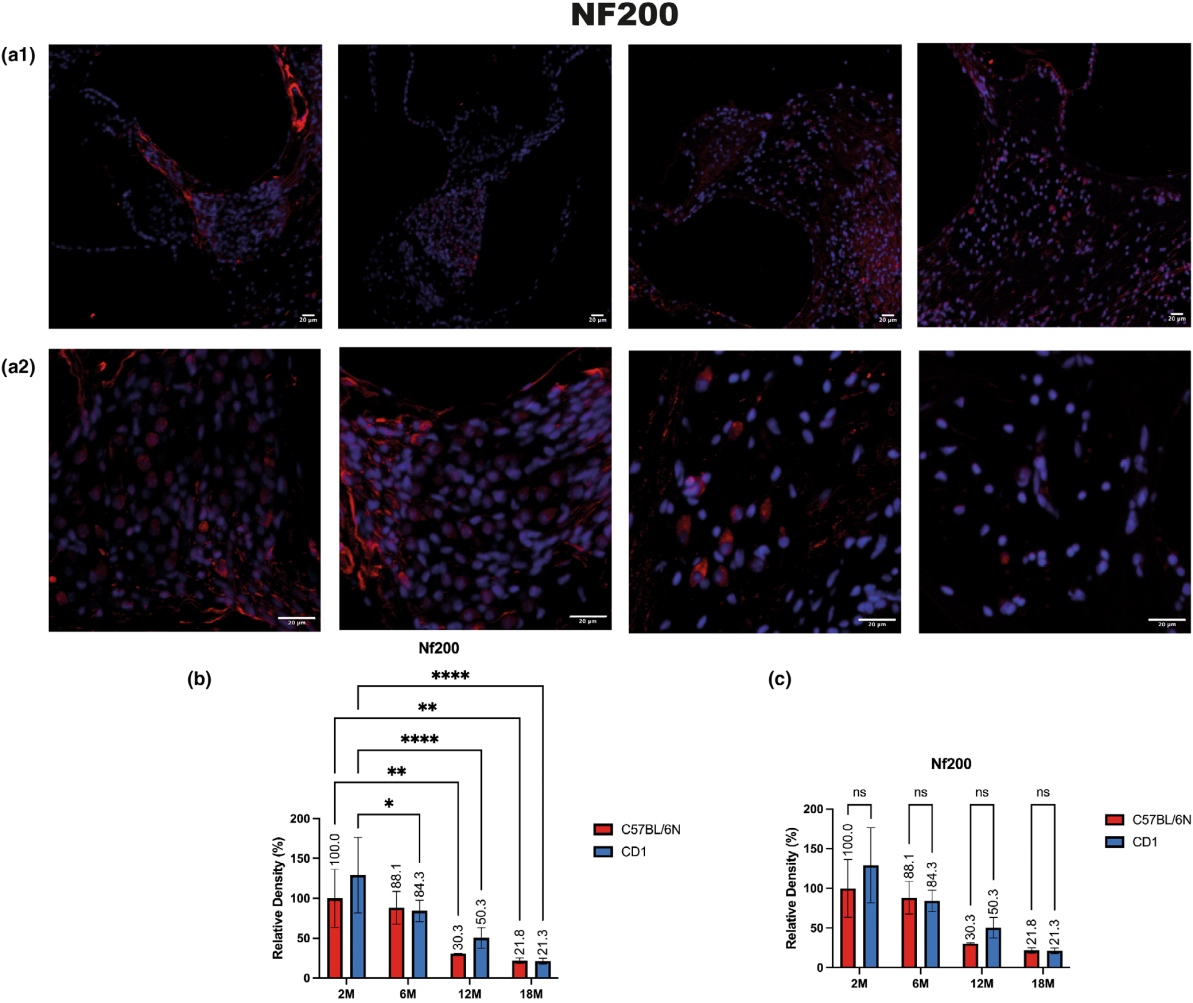
NF 200 in spiral ganglion neurons and quantitative analysis: (a1, a2) Immunofluorescence to NF200 in the spiral ganglia of C57BL/6N mice at 2, 6, 12, and 18 months of age at low (a1) and high (a2) magnification. The images are representative of both the strains. Scale bars 20 μm. (b, c) The histograms display the quantitative results of spiral ganglion neuron counts expressed as relative density of NF200 positive cells in the spiral ganglion area. (b) Comparisons between the different timepoints, 2, 6, 12 and 18 months for each strain. (c) Comparisons between the two strains, C57BL/6N versus CD1, at each timepoint. Differences between groups were analyzed by ANOVA followed by Tukey's HSD post‐hoc test. *p* values <0.05 (*), *p* values <0.01 (**), and *p* values <0.0001 (****) were considered statistically significant.

NF200 (Neurofilament protein NF200) signal distribution in spiral ganglion type 1 neurons appears both in the ganglion and along the fibers (Jyothi et al., 2010) (Figure 6a1,a2).

NF200 immunopositivity gradually decreased in both strains over aging (Figure 6b), in C57B/6 N, although not being significant, we observed a decrease of 11.9% between 2 and 6 months of age. The drop in signal density of 69.7% becomes more evident and statistically significant when considering animals at 2 months of age in comparison to 12 months old animals. Consistently, we observed a further decrease in tissue immunoreactivity density to anti NF200 Ab. The significant decrease in relative density was 78.7% in 18 months animals compared to that shown by 2 month mice. This corresponds to a further reduction of 8.5% detected between the ages of 12 and 18 months. In CD1, we observed a non‐significant reduction of 15.7%, in signal density at 6 months versus 2 months of age, which is in line with that reported inC57B/6 N. The difference in density starts to be significant with the comparison between 12 months of age and the 2 months, with a reported decrease of 49.7%. According to our data, the decrease of signal density consequent to the age‐related loss of SGNs seems to be more gradual in CD1 mice than in C57B/6 N mice. Despite the numeric dissimilarities we did not find statistically significant differences between the two strains over the entire lifespan (Figure 6c).

## 4 DISCUSSION

The generation of auditory sensation following sound perception depends on the integrity of the structures involved in the mechanical and electrical signal conduction, therefore, HCs and their stereocilia play a pivotal role in the mechanical conductivity of the peripheral auditory system. In the cochlea, the basilar and tectory membranes of the organ of Corti vibrate as sound waves pass through, causing the opening of the ionic channels of the stereocilia of the outer HCs. Through this mechanism the sound vibration is amplified and transmitted to the inner HCs. These effects trigger neurotransmitter release generating the electrical signals that, through SGNs, reach the superior olivary nucleus complex from which they are conveyed to the lateral thalamus and inferior colliculus to reach, at the end, the brain auditory cortex through the medial geniculate afferent fibers (Li et al., 2023). Within the SG, Type 1 neurons are myelinated, large, bipolar cells which establish single synapses with single inner HCs and represent 90%–95% of neurons constituting the spiral ganglion, unlikely Type 2 neurons which are small, unmyelinated cells which form multiple synapses with several outer HCs and correspond to just 5%–10% of the entire spiral ganglion neuron population (Carricondo & Romero‐Gómez, 2019; Wang, Xu, et al., 2022).

Therefore, the loss in sensory cells and SGNs microarchitecture and activity impairs auditory function.

The study of age‐related cochlear pathology in humans requires the use of animal models (Castaño‐González et al., 2024). In the translational setting, morphological mouse phenotyping is of utmost importance, especially in auditory research, where the mouse is a pivotal model organism (Bowl & Dawson, 2015; Castaño‐González et al., 2024), however, the inadequate description of experimental conditions contributes to poor reproducibility, making some results obtained in murine models untranslatable to humans (Justice & Dhillon, 2016). Aiming to characterize hearing loss over aging we selected an inbred strain, C57BL/6, featured by an accelerated presbycusis (Castaño‐González et al., 2024; Shone et al., 1991) and compared it against the outbred strain CD1. Both the strains, indeed, develop significant ARHL representing, therefore, valuable models to feature the morpho‐functional changes affecting the cochlea during aging. The selection of CD1 and C57BL/6 was based on their widespread use making them two of the most representative inbred and outbred strains used within the scientific community.

In general, studies on cochlea of adult and aged mice are few being the organ miniscule, the epithelium enclosed into a bony tissue, and the temporal bone extremely hard, all factors implying the risk to damage the organ during dissection (Fang et al., 2019; Montgomery & Cox, 2016). We set‐up an effective decalcification protocol to study the adult mouse cochlea maintaining the integrity of its anatomy and microarchitecture.

Montgomery and Cox as well as Fang and co‐workers report two valuable protocols in which the entire temporal bone was dissected and decalcified. In these two papers the procedure includes the retrieval of temporal bones of 9 and 2 month old mice and their decalcification using a 3.5% and a 4% EDTA solution respectively. Further, Montgomery and Cox report that EDTA may interfere with primary antibodies. In our study we effectively used a low EDTA concentration (2%) to decalcify the isolated cochlea instead of the entire temporal bone also guaranteeing the preservation of epitopes antigenicity in mice ranging from 2 to 18 months of age. We tested two concentrations of EDTA: 2% and 4%. The 4% concentration resulted in salt precipitation on the samples and, in some cases, loss of tissue integrity and macro‐architecture. The extensive washing steps required to remove the precipitated salts from the 4% EDTA solution led to additional tissue damage. Also, the extended incubation time, 14 days, contributed to ensure a gradual decalcification allowing to preserve tissue morphology.

Therefore, by sharing our protocol we provide a technical tool suitable to be exploited to generate further morphological data concerning adult mouse cochlea.

We focused our efforts on the comparison between two strains, CD1 and C57BL/6N, selected as representative models of inbred and outbred ones being the selection of the most appropriate strain type for any study an increasingly debated issue. Concerning the choice of C57BL/6 substrain, N instead of J, several reports are available on the use of both substrains in hearing research (Kendall & Schacht, 2014; Mekada & Yoshiki, 2021). However, sometimes the exact substrain is not even mentioned (Shone et al., 1991), thus making a comparative analysis more difficult. In the context of the comparison between outbred and inbred strains, a very interesting standpoint is that expressed by Tuttle et al.: an equal phenotypic variance shown by inbred and outbred strains may be linked to the wider impact of environmental variability on inbred animals and to a better predisposition of outbred ones to respond in a “finely tuned” manner to the different external stimuli (Biggers & Claringbold, 1954; Jensen et al., 2016; Tuttle et al., 2018). Based on this principle, the identification of strain‐specific markers in the case of apparently similar functional characteristics is crucial to select the ideal strain for a given study.

Aiming to characterize the auditory function over time in the two strains we measured the sound‐evoked auditory brainstem response at 2, 6, 12, and 18 months of age to evaluate auditory nerve function as well as the central auditory pathway in response to transitory sound. We chose as testing frequencies 8, 16, and 24 kHz representative of low, medium and high frequencies respectively, ranging the hearing ability of mice from 0.5 to 120 kHz with a higher sensitivity from 12 to24 kHz (Zheng et al., 1999). The results of the functional tests showed a decrease in auditory function in both strains and sexes over aging allowing to identify the transition from the young adult (6 months) to the adult age (12 months) as the life phase when the morpho‐functional decline starts.

Following, to feature the morphological sensory decline and the relevant differences in the degenerative process in the two strains, we performed histological and immunofluorescence analyses highlighting changes in the localization of key molecules in signal transduction at each timepoint in the same individuals previously subjected to the functional test. The morphological analysis was conducted on mixed‐sex animals, as the ABR did not show significant differences between the sexes in the two strains.

Specifically, to identify age‐related neurodegeneration indicators, we featured the localization of Gipc3, Myosin VIIa, and TMC1, in the HCs observing a decreasing trend in signal intensity and distribution. Also, to gain insights in the context of synaptopathies research, we performed NF200 immunolabeling of neural cytoskeleton to mark SGNs (Carricondo & Romero‐Gómez, 2019; Liberman & Kujawa, 2017).

As stated by Charizopoulou, Gipc3 is localized exclusively in cochlear HCs and SGNs where it seems to be included in a protein complex which regulates the trafficking of vesicles that vehiculate essential factors in signal detection and propagation. Therefore, in the organ of Corti it localizes in the body of hair cell and not in the stereocilia.

Myosin VIIa is an actin‐binding mechanoenzyme localized throughout the HCs, both in the stereocilia and body (Sirko & Kozlov, 2022; Wolfrum et al., 1998). As reported by El‐Amraoui and colleagues and Morgan and coworkers, in HCs Myosin VIIa seems to be involved in protein transport along the stereocilia actin filaments as well as in the preservation of stereocilia microarchitecture (Cosgrove & Zallocchi, 2014; Morgan et al., 2016). This protein is commonly used to selectively mark HCs since it is not significantly expressed in other cells of the organ of Corti (Zallocchi et al., 2012).

Regarding TMC1, a protein expressed in HCs from early stages of development (Li et al., 2019), Li and colleagues suggest a role as part of the mechanotransduction (MT) channel (Kawashima et al., 2011; Kim & Fettiplace, 2013; Pan et al., 2013). Interestingly, Pan et al. recently showed that TMC1 is the pore‐forming subunit of the MT channel and is included in macromolecular complexes of the HCs with other proteins required for mechanotransduction stability (Pan et al., 2018).

We observed a decrease in the expression of all the studied proteins over aging in the two strains. In particular, we detected a more intense signal and wider distribution of Myosin VIIa, at 6 months in comparison to 2 months of age in CD1 mice coherently with the ABR results. Therefore, by correlating morphological and functional data, we hypothesized the existence of potential «protective» mechanisms in CD1 mice based on the trend observed for Myosin VIIa at 6 months of age when the increased expression of this protein probably stabilizes the developing structure as, conversely, C57B/6 N mice display a significant decrease between 2 and 6 months of age. Therefore, we identified the different trend in CD1 as “protective” intending that at this life stage an improved stabilization, and, therefore, function, of cilia microarchitecture occurs. In the same strain we noticed, to a lesser extent, the same trend for Gipc3 potentially underlying a better acoustic signal acquisition and propagation in the HCs.

Concerning SGNs, playing a key role in the context of synaptopathies, by morphological analysis we observed that in C57BL/6N mice the loss of SGNs over aging seems to be more gradual than in CD1, likely due to the inbred nature of the strain. By NF 200 immunolabeling, instead, we detected a clear drop in signal occurrence throughout the spiral ganglion and in the number of type 1 SGNs.

In a future study we plan to apply an omics‐based approach as the studies tracking the aging process in the cochlea up until 18 months of age are few and none of them is focused on the comparison of different wild‐type mouse strains. Most studies, indeed, report omics analyses in disease and trauma models. For example, Sun and colleagues published a single‐cell transcriptomic atlas including 27 cochlear cell‐types in C57BL/6J mice of different ages ranging from 1 to 15 months thus featuring cochlear aging at high temporal and spatial resolution (Sun et al., 2023). In particular, the authors showed transcriptional variations in the stria vascularis intermediated cells and reported a key role of the endoplasmic reticulum (ER) chaperon protein HSP90AA1 in decreasing the level of ER stress‐induced impairment suggesting a target to delay aging‐related stria vascularis atrophy and, therefore, the advancement of ARHL.

Lao and colleagues, instead, examined and compared protein expression in the cochlear sensory epithelia in 1.5 months old and 6 months old C57BL/6J mice observing significant variation in the expression levels of 20 proteins included in the lower region of the cochlear sensory epithelia in older animals (Lao et al., 2024). This study reported, for the first time in AHRL studies, a variation in the expression of some proteins. In particular, the expression of 2 proteins, cartilage matrix protein and matrilin‐4, decreased in contrast with the increased expression of other 18 proteins, thus leading the authors to hypothesize that the abnormal expression of these proteins in the basal turn of the cochlea could be crucial in the early onset of ARHL.

Concerning aging associated metabolomic changes, Wang and colleagues compared cochlea metabolites between 1 month and 12 months old C57BL/6 mice showing significant changes in oxidative stress associated amino acids, in synaptic vesicle cycle of serotonin as well as in pantothenate and CoA biosynthesis. The authors hypothesized that the difference observed may be considered among the potential causes of presbycusis (Wang, Qiu, et al., 2022).

Based on the results reported in this study we provide potential useful insights to contribute to the characterization of sensorineural degeneration in the preclinical arena where data concerning mouse of 18 months of age are particularly valuable. The majority of results reported in the literature derive, indeed, from studies conducted on mice up to 1 year of age (Bowl & Dawson, 2015; Castaño‐González et al., 2024; Fang et al., 2019; Montgomery & Cox, 2016).

In conclusion, we showed a decrease of the sensorineural function over aging along with the development of key morpho‐functional variations at young adult‐adult transition in both strains, despite the different genetic background, as well as differences in potential strain‐specific «protective» mechanisms. In addition, we could argue that CD1, as outbred strain, might be more appropriate than inbred strains for a wide range of studies regardless of cases where genetic variability is expressly needed, such as toxicological studies, due to their more balanced response to environmental stimuli.

## 5 STUDY LIMITATIONS

Our study constitutes a preliminary step to link age‐related morphological and functional data gained in two commonly used wild type mice strains, widely spread as they stand but also largely employed in the generation of transgenic lines. This methodological approach sets the stage for future investigations including omics technologies to identify and characterize cell types as well as to explore gene expression patterns associated with cell function in such a complex organ. Undoubtedly, the use of omics techniques in anatomic studies offers several advantages over traditional histological and imaging methods. The omics technologies enable the simultaneous analysis of multiple molecular components of tissues and organs and are highly sensitive and specific revealing early signs of disease or abnormalities. Also, these techniques are quantitative, enabling the precise measurement of molecular changes in tissues and organs. However, despite these advantages, omics techniques have some limitations in morphological studies: one of the main challenges is that they often require the destruction of the sample, limiting their use for rare specimens. This is even more relevant when the target organ is as minuscule as the inner ear and, therefore, with limited availability. In view of this, future studies including omics data will be needed to unravel the cellular regulatory mechanisms. Specifically, starting from differential transcriptomics data and pathway analysis, our first milestone would be to overcome the limitations of looking at single‐gene expression to understand how genes interact with each other and how, within these gene networks, aging may induce dysregulations followed by degeneration and sensory decline. Pathway analysis will help to identify the biological processes or molecular mechanisms involved in cochlear degeneration over time. Degeneration stems from complex, multifactorial processes, therefore, the identification of the specific pathways affected at each time point may lead to track disease progression. Additionally, aiming our study to compare aging process in two strains with different genetic backgrounds, transcriptomics and pathway analysis, along with proteomics and metabolomics may highlight conserved and divergent mechanisms of hearing loss in the two strains. In the translational scenario, this aspect is particularly relevant in the outbred strain, where genetic variability better mimics the diversity of human populations in comparison to the inbred one. This wide set of data will provide broad insights into how cochlear degeneration might occur across species, including humans, and will help to select the most adequate mouse strain, inbred or outbred, to be included in studies addressed to develop novel therapeutic strategies.

## AUTHOR CONTRIBUTIONS

*Conceptualization*: Elena De Felice, Chiara Attanasio, Livia D'Angelo, Antonio Palladino. *Data curation*: Daniela Giaquinto, Marcello Raspa, Ferdinando Scavizzi. *Formal analysis*: Chiara Attanasio, Antonio Palladino, Elena De Felice. *Funding acquisition*: Elena De Felice, Marcello Raspac, Paolo de Girolamo. *Investigation*: Chiara Attanasio, Antonio Palladino, Chiara Peres, Camilla Anastasio. *Methodology*: Elena De Felice, Chiara Attanasio, Antonio Palladino, Daniela Giaquinto. *Project administration*: Elena De Felice, Marcello Raspa, Ferdinando Scavizzi. *Resources*: Elena De Felice, Marcello Raspa, Ferdinando Scavizzi. *Software*: Antonio Palladino. *Supervision*: Elena De Felice, Marcello Raspa, Paolo de Girolamo. *Validation*: Livia D'Angelo. *Visualization*: Carla Lucini, Paola Scocco. *Writing*—*original draft*: Chiara Attanasio. *Writing*—*review and editing*: Chiara Attanasio, Elena De Felice, Antonio Palladino.

## FUNDING INFORMATION

Financial support was provided by PRIN 2017 “Sensory Decay and Aging”‐ protocol number 2017FTJ5ZE, D.D.20/03/2019.

## CONFLICT OF INTEREST STATEMENT

All authors disclosed no conflicts of interest.

## Supporting information


**Data S1:** Supporting Information.

**Figure S1.** Auditory brainstem response results: The diagrams report the results of ABR tests for the two strains, CD1 and C57BL/6N, analyzed by sex, at each timepoints, 2, 6, 12, and 18 months of age. Data were grouped by type and frequencies of the acoustic stimuli (click threshold, 8000 Hz, 16,000 Hz, and 24,000 Hz).

**Figure S2.** Quantitative analysis of immunofluorescence: The histograms display the quantitative analysis of Gipc3, MyoVIIa, TMC1and NF200 Immunofluorescence. For the hair cell markers the results are expressed as relative fluorescence while for NF200 they are expressed as density of NF200‐positive cells. Differences between groups were analyzed by ANOVA followed by Tukey’s HSD post‐hoc test. *p* values <0.05 (*), *p* values <0.01 (**), *p* values <0.001 (***), and *p* values <0.0001 (****) were considered statistically significant.

## Data Availability

The data that support the findings of this study are available on request from the corresponding author. The data are not publicly available due to privacy or ethical restrictions.
