# Supplementary figures and images for: Morphological phenotyping of the aging cochlea in inbred C57BL/6N and outbred CD1 mouse strains
Source: Aging Cell. 2024 Oct 31;24(1):e14362. doi: 10.1111/acel.14362 (PMC11709085; doi:10.1111/acel.14362)

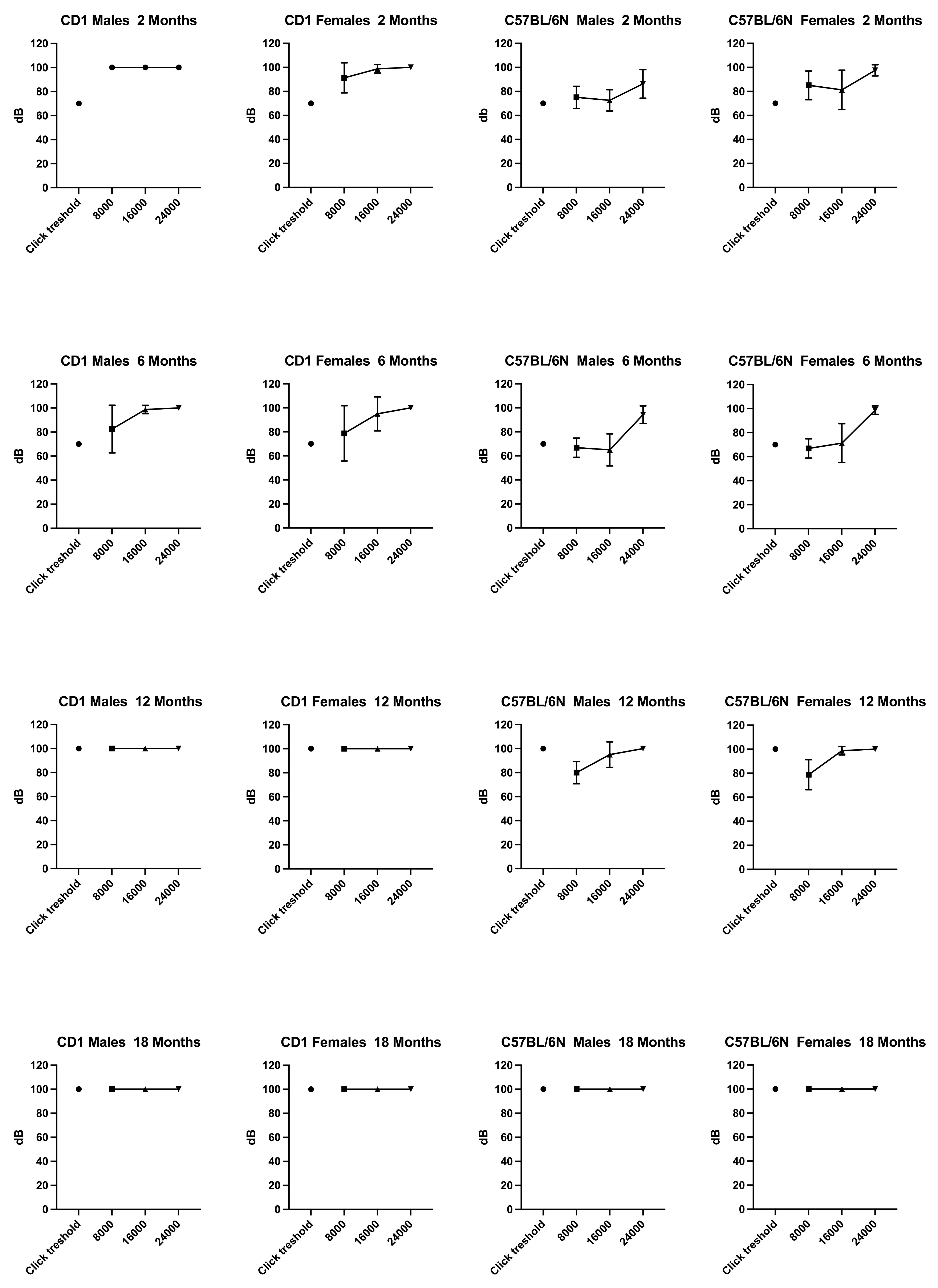

Supplement: Supplementary file 2 — Figure S1. Auditory brainstem response results: The diagrams report the results of ABR tests for the two strains, CD1 and C57BL/6N, analyzed by sex, at each timepoints, 2, 6, 12, and 18 months of age. Data were grouped by type and frequencies of the acoustic stimuli (click threshold, 8000 Hz, 16,000 Hz, and 24,000 Hz). [file ACEL-24-e14362-s003.jpg]

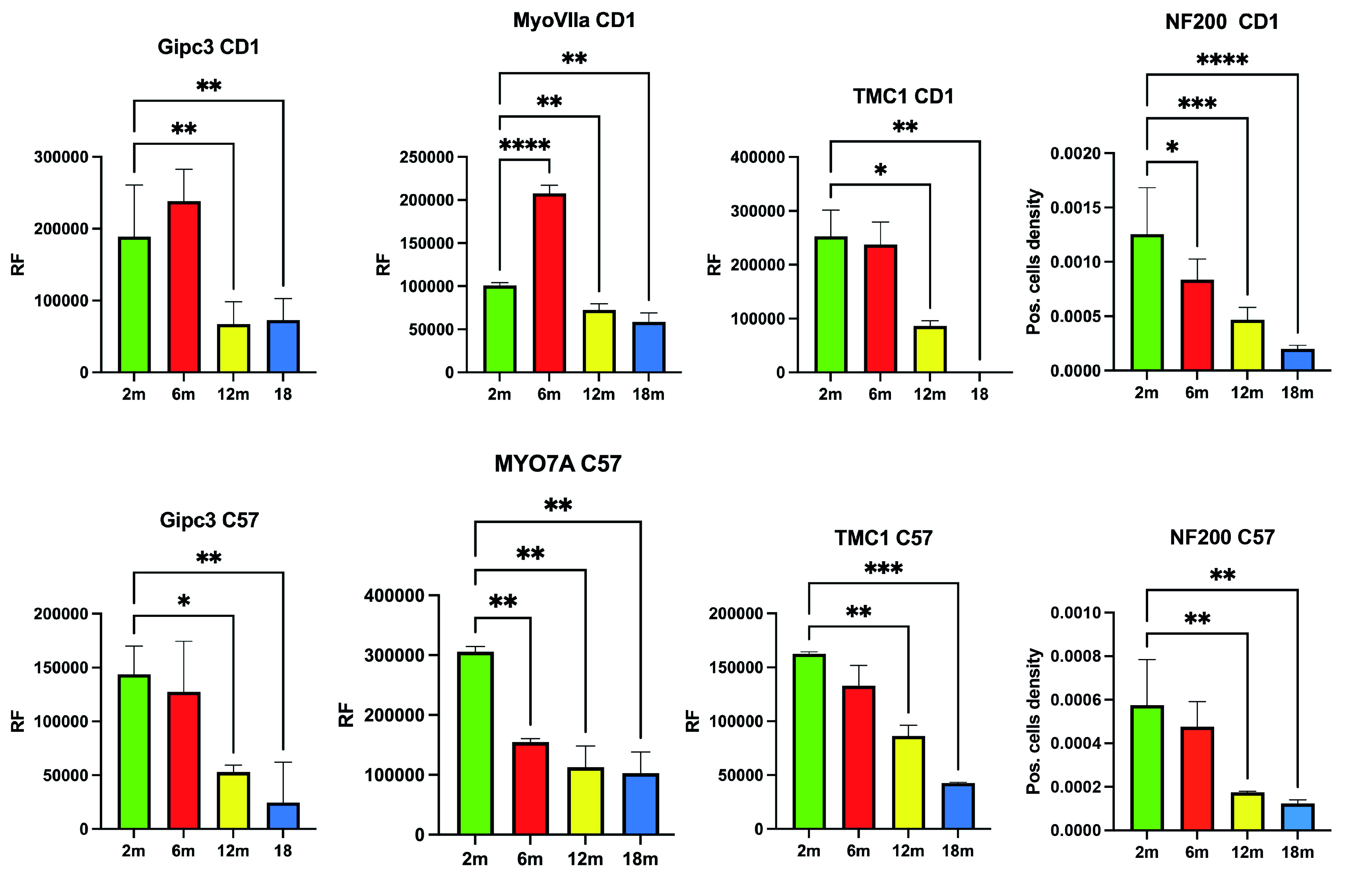

Supplement: Supplementary file 3 — Figure S2. Quantitative analysis of immunofluorescence: The histograms display the quantitative analysis of Gipc3, MyoVIIa, TMC1and NF200 Immunofluorescence. For the hair cell markers the results are expressed as relative fluorescence while for NF200 they are expressed as density of NF200‐positive cells. Differences between groups were analyzed by ANOVA followed by Tukey’s HSD post‐hoc test. p values <0.05 (*), p values <0.01 (**), p values <0.001 (***), and p values <0.0001 (****) were considered statistically significant. [file ACEL-24-e14362-s001.jpg]
